# Agents for the Removal of Stains

**Published:** 1870-02

**Authors:** 


Agents for the Removal of Stains.—Dr. Bottger gives a recapitulation of the most approved agents for removing stains from linen and table service. To remove red wine and berry stains cover up the spot with pulverized tartaric acid, and wash out with Javille water. To remove silver stains a cautious use of a warm concentrated solution of cyanide of potassium is the best reagent. For ink spots use a concentrated solution of bin-oxalate of potash. To remove iron rust stains, immerse the spot in a boiling hot saturated solution of bin-oxalate of potash, and afterward strew over it fine tin powder.—*Jour. of Applied Chemistry.*

